# Development of immune‐related cell‐based machine learning for disease progression and prognosis of alcoholic liver disease

**DOI:** 10.1002/ctm2.1322

**Published:** 2023-06-28

**Authors:** Xu Zhang, Xuezhe Feng, Qian Huai, Cheng Zhu, Yingyin Sun, Xiaolei Li, Hanren Dai, Honglin Wang, Hua Wang

**Affiliations:** ^1^ Department of Oncology First Affiliated Hospital of Anhui Medical University Hefei China; ^2^ Department of Stomatology First Affiliated Hospital of Anhui Medical University Hefei China; ^3^ Department of Microscopic Orthopedics the Hefei Second People's Hospital & Hefei Affiliated Hospital of Anhui Medical University Hefei China

Dear Editor,

Immune‐related cell (IRCs)‐based machine learning (ML) models, including random forest (RF), multilayer perceptron (MLP), generalised linear model (GLM) and gradient boosting machine (GBM), have shown great performance in the estimation of alcoholic liver disease (ALD).

Pathological biopsy of the liver is considered the most reliable method for determining the diagnosis and evaluating the staging and prognosis of liver disease, but liver biopsy can cause complications and therefore lacks specific noninvasive diagnostic biomarkers.^1^ IRCs are biomarkers of systemic inflammation, and identifying changes in IRCs associated with ALD will contribute to improving the diagnosis of ALD.^2^, ^3^ However, current biomarker discoveries are usually focused on an individual biomolecule, resulting in low clinical applicability,^4^, ^5^ and few studies have investigated diagnostic value of IRCs in ALD. Therefore, there is urgent ongoing research exploring an accurate and sensitive noninvasive test that is low cost and low risk. Recently, ML has been widely used in biomedical research and disease diagnosis, and ML is a promising effective method in the identification of hepatic fibrosis and cirrhosis.^3^, ^4^ However, no studies have investigated the role of ML model, especially IRC‐based ML model, in the diagnosis, disease progression and prognosis of ALD.

In this study, we developed IRC‐based ML models to assess ALD progression and prognosis. Overall, 207 ALD patients (including alcoholic fatty liver (AFL) and alcoholic cirrhosis (ALC)) and 234 healthy controls (HCs) were included (Figure 1A and Table 1). ML had great performance in the estimation of ALD. To explore the role of IRCs in disease onset, we compared the differences between ALD and HCs. Gender, age, MCV, platelets, RBCs, neutrophil/lymphocyte ratio (NLR) and monocyte/neutrophil ratio (MNR) were included in the nomogram using LASSO regression (Figure 1B–D). Decision curve analysis (DCA) curve found that the net benefit of the nomogram was 0.03 to 0.99 (Figure 1C), suggesting that the predictive ability and accuracy of model fitting are high. Here, we introduced the findings of the RF model, as it had the greatest performance (Figure 1E–H and Table S1). AUCs of PR and ROC were 0.9982 and 0.9975 in the training set, and 0.9984 and 0.9978 in the testing set, respectively (Figure 1E and F and Table 1). In addition, the evaluation of ML (i.e., root mean square error (RMSE) and mean square error (MSE)) is shown in Table S2, indicating that the ML models are robust and reliable. These results suggested that ML models, especially the RF model, not only have high precision but also have high predictability. We conducted additional ML models for ALD using variables not screened by least absolute shrinkage and selection operator (LASSO) regression as sensitivity analyses (Figure 1I–L). Results were consistent with regression screening for most outcomes, which means that our results are robust and that we can obtain good predictions by using several important variables instead of all variables.

**FIGURE 1:**
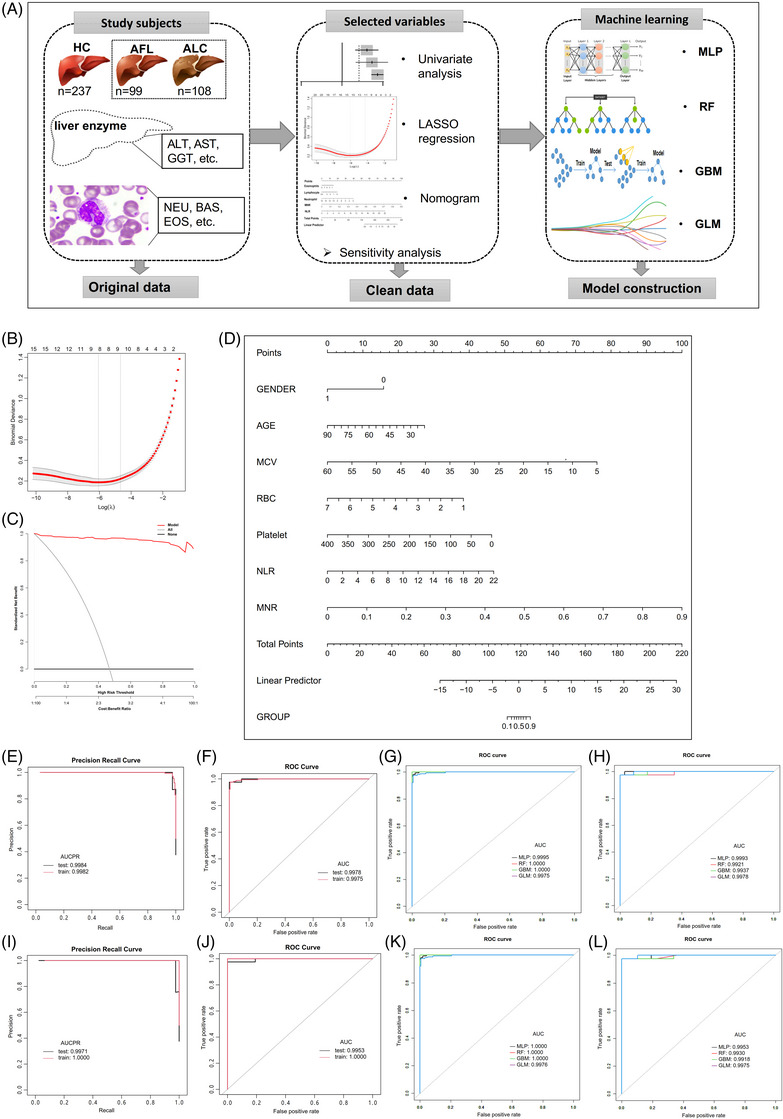
Machine learning performance in the estimation of ALD. (A) Immune‐related cells (IRCs) were measured in patients with ALD and healthy controls as the original data. Significant IRCs in the univariate analysis were analysed by LASSO regression as clean data, and a nomogram was used to visualise the results. In addition, sensitivity analysis was performed for all IRCs. Four ML models, including MLP, RF, GBM, and GLM, based on significant IRCs were constructed. (B) LASSO regression profiles of 7 parameters. (C) DCA curve for assessing the predictive ability and accuracy of the nomogram. (D) Nomogram for predicting ALD probability. (E) AUCPR of the best ML model. (F) AUCs of the best ML model. (G) AUCs of the four ML models in the training set. (H) AUCs of the four ML models in the testing set. (I) AUCPR of the best ML model in the sensitivity analysis. (J) AUCs of the best ML model in the sensitivity analysis. (K) AUCs of the four ML models in the training set of the sensitivity analysis. (L) AUCs of the four ML models in the testing set of the sensitivity analysis.

**TABLE 1:** Performance of ML models in the estimation of disease diagnosis and progression.

	Accuracy		AUC		AUCPR		Sensitivity		Specificity		PPV		NPV	
	Train	Test	Train	Test	Train	Test	Train	Test	Train	Test	Train	Test	Train	Test
**ALD onset**														
**ALD vs. HC**														
MLP	0.9880	0.9908	0.9995	0.9993	0.9996	0.9994	0.9940	0.9756	0.9819	1.0000	0.9821	1.0000	0.9939	0.9855
RF	1.0000	0.9908	1.0000	0.9921	1.0000	0.9949	1.0000	0.9756	1.0000	1.0000	1.0000	1.0000	1.0000	0.9855
GBM	0.9970	0.9908	1.0000	0.9937	1.0000	0.9966	0.9940	0.9756	1.0000	1.0000	1.0000	1.0000	0.9940	0.9855
GLM	0.9849	0.9908	0.9975	0.9978	0.9982	0.9984	0.9759	0.9756	0.9940	1.0000	0.9939	1.0000	0.9763	0.9855
**ALD progression**														
**AFL vs. HC**														
MLP	0.9960	1.0000	0.9990	0.9999	0.9990	1.0000	0.9882	1.0000	1.0000	1.0000	1.0000	1.0000	0.9940	1.0000
RF	1.0000	1.0000	1.0000	0.9997	0.9902	1.0000	1.0000	1.0000	1.0000	1.0000	1.0000	1.0000	1.0000	1.0000
GBM	0.9960	0.9890	0.9988	0.9999	1.0000	1.0000	0.9882	0.9565	1.0000	1.0000	1.0000	1.0000	0.9940	0.9855
GLM	0.9920	1.0000	0.9946	0.9999	0.9979	1.0000	0.9882	1.0000	0.9940	1.0000	0.9882	1.0000	0.9940	1.0000
**ALC vs. HC**														
MLP	0.9835	0.9889	0.9988	0.9995	0.9964	0.9973	0.9610	0.9545	0.9940	1.0000	0.9867	1.0000	0.9821	0.9855
RF	1.0000	0.9889	1.0000	0.9996	1.0000	0.9945	1.0000	0.9545	1.0000	1.0000	1.0000	1.0000	1.0000	0.9855
GBM	1.0000	0.9778	0.9999	0.9995	1.0000	0.9990	1.0000	0.9091	1.0000	1.0000	1.0000	1.0000	1.0000	0.9714
GLM	0.9835	0.9778	0.9946	0.9996	0.9924	1.0000	0.9610	1.0000	0.9940	1.0000	0.9867	1.0000	0.9821	0.9714
**ALC vs. AFL**														
MLP	0.8125	0.6190	0.8543	0.6897	0.8777	0.6995	0.8354	0.5862	0.7846	0.6471	0.8250	0.5862	0.7969	0.6471
RF	0.9792	0.5873	1.0000	0.7307	1.0000	0.6924	1.0000	0.7586	0.9538	0.4412	0.9634	0.5366	1.0000	0.6818
GBM	0.8542	0.7143	0.9274	0.7566	0.9399	0.7833	0.8861	0.6207	0.8154	0.7941	0.8537	0.7200	0.8548	0.7105
GLM	0.7569	0.5873	0.8249	0.7089	0.8528	0.7567	0.8987	0.6207	0.5846	0.5588	0.7245	0.5455	0.8261	0.6333
**ALD prognosis**														
**MDF (≥32.2 vs. < 32.2)**														
MLP	0.9653	0.7302	0.9257	0.8413	0.9954	0.8884	0.9592	0.7619	0.9783	0.6667	0.9895	0.8205	0.9184	0.5833
RF	0.7014	0.6667	1.0000	0.7902	1.0000	0.8854	1.0000	1.0000	0.6520	0.0000	0.6950	0.6667	1.0000	NA
GBM	0.7292	0.6984	0.8281	0.8033	0.8878	0.9298	0.9796	0.9762	0.1957	0.1429	0.7218	0.6949	0.8182	0.7500
GLM	0.7292	0.6825	0.6697	0.7823	0.7921	0.8880	1.0000	0.9524	0.1522	0.1429	0.7153	0.6897	1.0000	0.6000
**MELD (≥20 vs. < 20)**														
MLP	0.9306	0.8571	0.9013	0.6931	0.6903	0.5183	0.5882	0.3000	0.9764	0.9623	0.7692	0.6000	0.9466	0.8793
RF	1.0000	0.8413	1.0000	0.7151	1.0000	0.5231	1.0000	0.4000	1.0000	0.9245	1.0000	0.5000	1.0000	0.8909
GBM	0.9861	0.8571	0.9977	0.7057	0.9831	0.4613	0.9412	0.2000	0.9921	0.9811	0.9412	0.6667	0.9921	0.8667
GLM	0.8889	0.8413	0.8652	0.7821	0.6762	0.4724	0.7059	0.5000	0.9134	0.9057	0.5217	0.5000	0.9587	0.9057

ALD, alcoholic liver disease; ALC, alcoholic liver cirrhosis; AFL, alcoholic fatty liver; AUC, area under curve for receiver operating characteristic; AUCPR, area under curve for precision‐recal; MLP, multilayer perceptron; GBM, gradient boosting machine; GLM, generalised linear model; HCs, healthy controls; RF, random forest; MDF, Maddrey's discriminant function; MELD, model for end‐stage liver disease; NPV, negative predictive value; PPV, positive predictive value.

To further investigate the role of IRCs in the disease progression of ALD, we conducted subgroup analyses in AFL and ALC patients. First, 13 variables may be potential risk factors for AFL patients, and 8 variables including gender, basophils, MCV, lymphocytes, neutrophils, platelets, NLR and MNR were included. The RF model had the greatest performance, and AUCs of PR and ROC were 0.9984 and 0.9990 in the training set, and 1.0000 and 1.0000 in the testing set, respectively (Figure 2A–G and Table 1). Second, 17 variables may be related to ALC patients, and 10 variables were eventually left in the LASSO regression and nomogram (Figure 2H–N), that is, age, gender, MCV, lymphocyte, platelet, RBC, platelet/lymphocyte ratio (PLR), platelet/neutrophil ratio (PNR), MNR and NLR. The RF model had the greatest performance (Figure 2M and N and Table 1). Finally, 13 variables may be related to disease progression when comparing ALC with AFL, and 11 variables were eventually left in the LASSO regression and nomogram. The RF model had the greatest performance (Table 1 and Figure S1).

**FIGURE 2:**
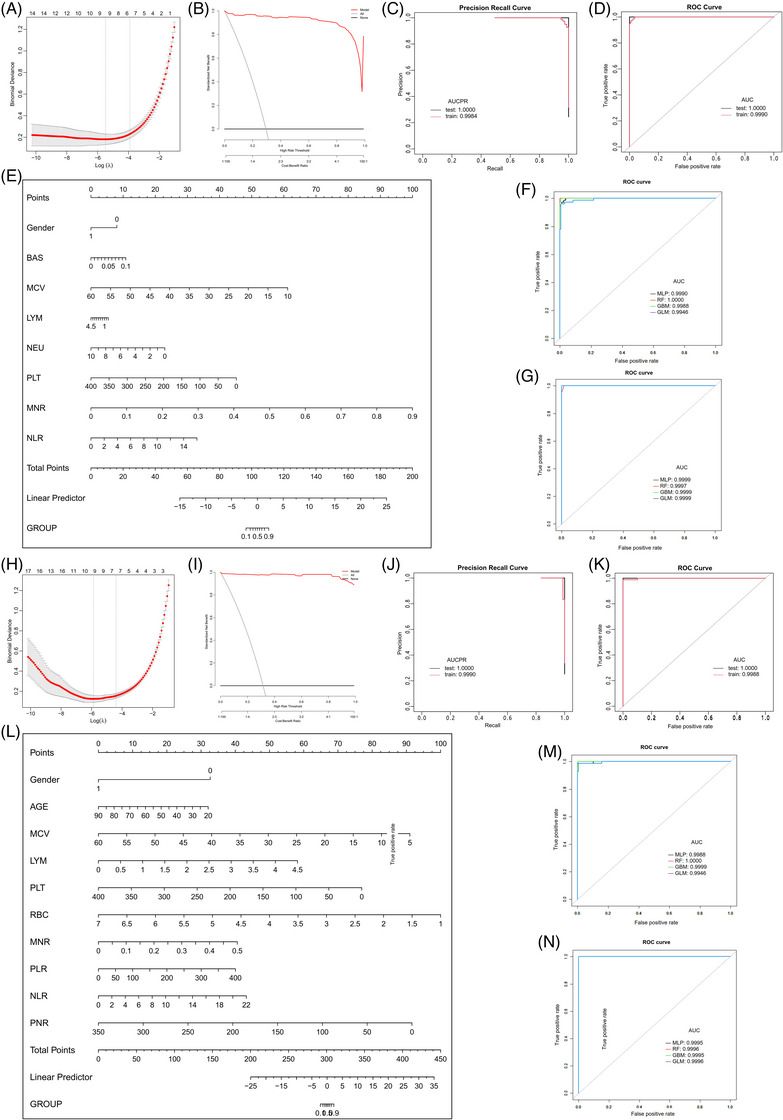
Machine learning performance in the estimation of AFL and ALC. (A) LASSO regression profiles of 8 parameters in patients with AFL. (B) DCA curve for assessing the predictive ability and accuracy of the nomogram in patients with AFL. (C) AUCPR of the best ML model in patients with AFL. (D) AUCs of the best ML model in patients with AFL. (E) Nomogram for predicting AFL probability. (F) AUCs of the four ML models in the AFL training set. (G) AUCs of the four ML models in the AFL testing set. (H) LASSO regression profiles of 10 parameters in patients with ALC. (I) DCA curve for assessing the predictive ability and accuracy of the nomogram in patients with ALC. (J) AUCPR of the best ML model in patients with ALC. (K) AUCs of the best ML model in patients with ALC. (L) Nomogram for predicting ALC probability. (M) AUCs of the four ML models in the ALC training set. (N) AUCs of the four ML models in the ALC testing set.

To further explore the role of IRCs in the prognosis of ALD, MELD (model for end stage liver disease) and MDF (Maddrey's discriminant function) score‐based ML were used to predict disease severity. MELD‐associated LASSO regression found that basophils, MCV, neutrophils, RBC and white blood cells (WBCs) were potential prognostic factors (Figure 3A–G). The RF model had high performance (Figure 3F andG and Table 1). In addition, MDF‐associated LASSO regression found that eosinophils, platelets, RBCs, PMR and PNR were potential prognostic factors (Figure 3H–N). The MLP model had high performance (Figure 3M andN and Table 1). These significant IRCs and the best model of MDF differ from the MELD‐based model. One possible explanation is that the evaluation method is different between the two scores.

**FIGURE 3:**
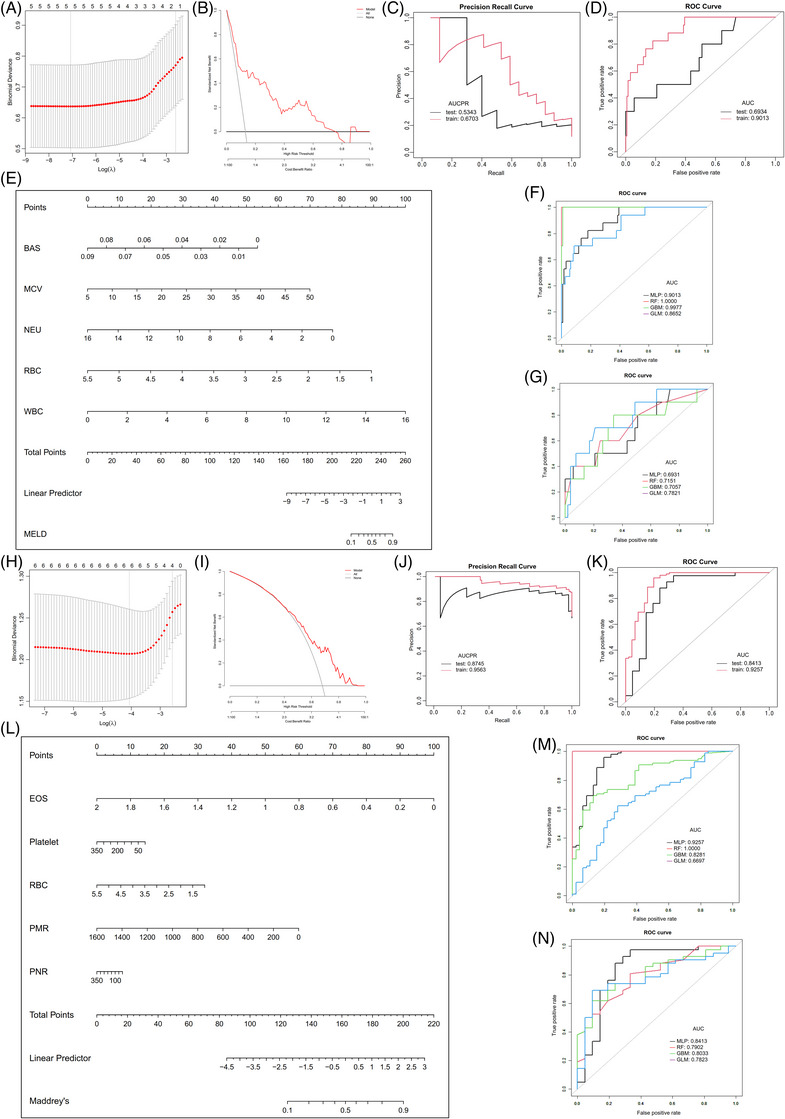
Machine learning in the estimation of ALD prognosis (MELD and MDF). (A) MELD‐based LASSO regression profiles of 5 parameters. (B) MELD‐based DCA curve for assessing the predictive ability and accuracy of the nomogram. (C) AUCPR of the best MELD‐based ML model. (D) AUCs of the best MELD‐based ML model. (E) Nomogram for predicting MELD‐based ALD probability. (F) MELD‐based AUCs of the four ML models in the training set. (G) MELD‐based AUCs of the four ML models in the testing set. (H) MDF‐based LASSO regression profiles of 5 parameters. (I) MDF‐based DCA curve for assessing the predictive ability and accuracy of the nomogram. (J) AUCPR of the best MDF‐based ML model. (K) AUCs of the best MDF‐based ML model. (L) Nomogram for predicting MDF‐based ALD probability. (M) MDF‐based AUCs of the four ML models in the training set. (N) MDF‐based AUCs of the four ML models in the testing set.

ML represents the future of clinical medicine and will be implemented in healthcare systems to facilitate early identification of target populations. We used four ML models to predict ALD, which were constructed based on IRCs that have significant roles in liver injury, fibrogenesis and regeneration.^6^ Different IRCs play different leading roles in disease progression and prognosis, which means that appropriate cells are needed to assess the different stages of the disease. Neutrophils, the most common components of circulating WBCs, are implicated in the immune pathogenesis of ALD with complex and multifaceted properties, that is, neutrophils directly cause hepatocyte injury and liver inflammation in ALD, while neutrophil‐induced cytokines have a protective role in inflammation and liver repair.^7^, ^8^ In addition to neutrophils alone, the effects of neutrophil and immune cell interaction (e.g., PNR, MNR and NLR) were also relevant features of the ML models. Platelet interactions with IRCs, especially neutrophils, have been extensively investigated in a variety of diseases^6^, ^9^ except liver diseases. Future studies are highly recommended to explore the diversity and complexity of neutrophil functions to formulate targeted interventions and treatments for ALD. In addition, systemic inflammation is often accompanied by changes in RBCs. For example, RBCs increase the risk of liver disease, and RBC count is related to fatty liver index and disease progression.^10^

In conclusion, IRCs and their interactions are of great importance in ALD, and IRC‐based ML models, especially the RF model, are accurate and inexpensive tools for identifying the progression and prognosis of ALD. This study could be used as a basis for the development of ML models for disease prediction.

## CONFLICT OF INTEREST STATEMENT

The authors declare no conflicts interests.

## FUNDING INFORMATION

XZ is supported by the National Natural Science Foundation of China (82100628), the Natural Science Foundation of Anhui Province (2108085QH313), the Postdoctoral Research Foundation of China (2021M700183B496). YS is supported by the National Natural Science Foundation of China (82100627) and the Natural Science Foundation of Anhui Province (2108085QH311).

## Supporting information

Supporting information

## Data Availability

Raw data are available on reasonable request.
