# Supplementary material for: Development of immune‐related cell‐based machine learning for disease progression and prognosis of alcoholic liver disease
Source: Clin Transl Med. 2023 Jun 28;13(7):e1322. doi: 10.1002/ctm2.1322 (PMC10307989; doi:10.1002/ctm2.1322)
Supplement: Supplementary file 1 — Supporting information [file CTM2-13-e1322-s001.docx]

# Supplementary materials

# Materials and methods

## Human subjects

This retrospective case-control study was conducted in the Chinese Han population, and a total of 441 human subjects (aged 18-65 years) including 207 patients with alcoholic liver disease (ALD) and 234 healthy controls (HCs) were included. Patients with ALD were recruited from the Department of Gastroenterology, First Affiliated Hospital of Anhui Medical University, and met the modified 2010 EASL ALD Clinical Practical Guidelines of the Chinese Medical Association (1).

A diagnosis of patients with ALD had average daily ethanol consumption of 40 grams per day for males and 20 grams per day for females, with a duration of at least 5 years. Serological aspartate transaminase (AST), alanine transaminase (ALT), and gamma-glutamyl transferase (γ-GT) levels increased, especially AST/ALT > 2. Computed tomography (CT) or Ultrasound examination of the liver showed fatty liver or cirrhosis. Patients with viral hepatitis (e.g., hepatitis B virus (HBV) and hepatitis C virus (HCV)), drug-induced liver injury, autoimmune liver disease and malignant diseases (e.g., tumor) were excluded. HCs were included from the Center of Physical Examination, First Affiliated Hospital of Anhui Medical University. HCs had normal hepatic panel, no previous history of ALD, and the self-reported drinking level did not exceed the upper limit. In addition, there are no symptoms of hepatosplenomegaly or liver diseases (e.g., HBV and HCV, etc.). All subjects must have complete data for analysis, and they are unrelated and had written informed consent. This study was supported by the Clinical Hospital Research Ethics Committee of the First Affiliated Hospital of Anhui Medical University (ethics number: 5101229).

## Complete blood count test and biochemical assays

Peripheral blood was collected from patients with ALD and HCs using ethylene diamine teraacetic acid (EDTA) anticoagulant tubes, and serum samples were prepared by centrifugation (1500rpm for 10 min). Routine blood chemistry analyses, including liver enzymes (e.g., ALT and AST) and immune-related cells (IRCs, e.g., neutrophils, lymphocytes, and monocytes), were carried out in the HITACHI Automatic Aralyzer using standard clinical laboratory methods. We assessed disease severity, including maddrey discriminant function (MDF) and model for end-stage liver disease (MELD), by calculating biochemical parameters.

In addition, PLR, PMR, and PNR were calculated as platelet count divided by lymphocyte count, monocyte count, and neutrophil count, respectively. MLR and MNR were calculated as monocyte count divided by lymphocyte count, and neutrophil count, respectively. NLR was calculated as neutrophil count divided by lymphocyte count.

## Machine learning models

Recently, Chang et al. used random forest (RF), artificial neural network (ANN), and logistic regression (LR) models to investigate clinical stages of non-alcoholic fatty liver disease (NAFLD) and NAFLD-related cirrhosis (2). Therefore, we chose four common ML models, including multilayer perceptron (MLP, also known as a fully connected deep neural network), random forest (RF), gradient boosting machine (GBM), and generalized linear model (GLM), to predict patients with ALD. Firstly, MLP is a form neural network model. Secondly, GLM is an extension of a linear model that can be used to explore relationship between dependent and independent variables. Compared to the LR, GLM can handle the independent variables that do not conform to the normal distribution hypothesis, and can be applied to categorical and continuous variables. Finally, we added an additional model, i.e., gradient boosting machine (GBM) model (it is also well-accepted model). GBM, an ensemble learning algorithm, improves the accuracy of a model by combining multiple weak prediction models into one strong prediction model with a small error. In addition, categorical and numerical values can be included in the GBM model at the same time.

MLP, also known as artificial neural network (ANN), is a generalization of the single-layer perceptron, the most important feature is that it has multiple hidden layers in addition to input layer and output layer. The first layer of the MLP is called the input layer, the middle layer is the hidden layer, and the last layer is the output layer. MLP does not specify the number of hidden layers, so the appropriate number of hidden layers can be selected according to the actual processing needs, and there is no limit to the number of neurons in each layer of the hidden layer and the output layer.

Three nodes in the MLP model:

1. Input node: Also known as input layer, input nodes provide information from the external world. In the input node, no calculation is performed and only information is transmitted to the hidden node.

2. Hidden node: Also known as hidden layers, hidden nodes have no direct connection to the external world. These nodes perform calculations and transmit information from the input node to the output node. Although a GLP has only one input layer and one output layer, there may be multiple hidden layers.

3. Output node: Also known as output layers, output node are responsible for computing and transmitting information from the network to the external world.

Random forest (RF) is a ML algorithm for classification, regression and feature selection. It is a comprehensive learning that combines multiple decision trees to make more accurate predictions. In random forests, each decision tree is trained on a randomly selected data subset and a randomly selected feature subset, which helps to reduce overfitting and improve the accuracy of model. When making predictions, each tree gives a classification (i.e., the tree “votes” for one class), and then the final forest chooses is selected from the most votes.

Each tree in the forest is grown as follows:

1. If there are N samples in the training set, then sampling N with replacement at random from the original data. The samples are used for growing the tree in the training set.

2. If there are M input features, m features are selected out of the M (m<<M) at each node, and choose the best splitting point among the m features for splitting the node.

3. Every tree grows as much as possible without pruning.

GLM, an extension of a linear model, is used to explore relationship between dependent and independent variables. Compared to the LR, GLM can handle the independent variables that do not conform to the normal distribution hypothesis, and can be applied to categorical and continuous variables. GLM involves three elements including random component, system component, and link function.

1. System component is used to explain the research phenomenon in the regression.

2. Random component is used to predict the unknown shape, i.e., the shape of the response variable.

3. Link function describes the relationship between random component and system component.

GBM, a boosting algorithm, is composed of multiple learners. The main idea of gradient boosting is to generate multiple weak learners sequentially, and the goal of each weak learner is to fit the loss function of the previously accumulated model, thus the cumulative model loss after the addition of the weak learner is reduced. This process is repeated multiple iterations until the error is minimized or the maximum number of iterations is reached. Gradient lifting involves three elements including optimization of loss function, prediction of a week learner, and an addition model with a weak learner to minimize loss function.

## Feature selection and model training

Ultimately, 441 human subjects including 207 patients with ALD and 234 HCs were included with 22 features (Supplementary Table 1). Of the 22 variables, two were demographic characteristics (i.e., gender and age), six liver enzymes were related to disease diagnosis (i.e., ALT, AST, ALP, γ-GT, and AST/ALT ratio) (1, 3, 4), and 15 were IRCs (i.e., basophils, eosinophil, mean corpuscular volume (MCV), red blood cell (RBC), white blood cell (WBC), lymphocyte, monocyte, neutrophils, and platelet) and their ratios (i.e., PMR, MLR, MNR, PLR, NLR, and PNR), which were involved in the pathogenesis of the disease (5-7). Subsequently, a random 75% of dataset was used for ML training models, and the remaining 25% was used for ML testing models. Even though the model being divided into 75% training sets and 25% testing sets, dataset is still balanced and there is no missing value. Finally, we fit MLP, RF, GBM, and GLM to the training sets, and each model was used to generate predictions for the testing data. Meanwhile, disease progression (AFL vs. HC, ALC vs. HC, and ALC vs. AFL) and prognosis (MDF and MELD scores) were also used to generate predictions.

**Evaluation of model fitting effect**

In this study, we used two methods to determine the model fitting effect including using cross validation and observing the variation of errors between the two groups. Firstly, cross-validation is a tool to evaluate model performance and generalization ability. It will obtain a stable model performance by dividing data into training set and testing set, and repeating random division many times. If the model performs well on the training set but poorly on the testing set (i.e., the model does not perform well on the prediction of unknown samples and has poor generalization ability.), there may be an overfitting.

Secondly, observing the training-related and testing-related errors during the training process. Overfitting may occur if the training error continues to decline while the testing error continues to rise. The overfitting representation model performs well on training data, but has poor generalization ability on new data. In our models, we observed the changes of mean square error (MSE) and root mean square error (RMSE) in the training set and testing set. In addition, we used LASSO (least absolute shrinkage and selection operator) regression to address the overfitting by introducing regularization.

**Decision curve analysis (DCA) curve and net benefit**

Decision curve analysis (DCA) is a tool used to evaluate the benefits of diagnostic testing for many patients with undertreated and overtreated risk to facilitate decision making about test selection and use. The net benefit is calculated by the difference between the expected benefit and the expected harm associated with each proposed treatment and testing strategy. The expected benefit is expressed by the number of patients who have the disease and will be treated using the proposed strategy (true positives). The expected harm is represented by the number of disease-free patients who receive the wrong treatment (false positive) multiplied by a weighting factor based on the probability of the patient's threshold. The weighting factor captures the patient’s values regarding the risks of undertreatment and overtreatment. Specifically, the false-positive rate is multiplied by the ratio of the threshold probability divided by 1 - the threshold probability. Graphically, DCA is represented as a curve with the benefit score on the vertical axis and the probability thresholds on the horizontal axis. A curve is drawn for each possible approach used to establish a diagnosis. One line (the black line) shows what happens when the treatment is never given (i.e., no net benefit), and another curve (the gray line) shows that all patients receive treatment regardless of test results.

**Net benefit calculation:**

Net benefit = true positive rate - (false positive rate x weighting factor).

Weighting factor = (Threshold probability) / (1 - threshold probability).

## Statistical analysis

The Kolmogorov-Smirnov test was used for the normal distribution of continuous variables, and the independent T-test and Mann-Whitney U test were used for data comparison between patients with ALD and HC as appropriate. Chi-square test was used to compare the differences of categorical variable (i.e., gender). Multivariate analysis was conducted for variables that were statistically significant in the univariate analysis.

Considering the existence of multicollinearity between independent variables (i.e., there may be correlations between different IRCs) in multivariate analysis, LASSO (least absolute shrinkage and selection operator) regression was used to address it. More importantly, no matter whether the dependent variable is continuous variable or categorical variable, it can be modeled and predicted by LASSO regression. In addition, LASSO regression can address the overfitting by introducing regularization. Therefore, we conducted LASSO regression to filter independent risk of IRCs. Subsequently, nomogram was used to visualize prediction, and the predictive ability and accuracy of nomogram were assessed by decision curve analysis (DCA) curve.

Four ML models (previously described) were conducted based on significant IRCs, we also used all IRCs (i.e., without LASSO regression screening variables) for conducting ML as sensitivity analyses. Receiver operating characteristic (ROC) curve and precision-recall (PR) curve and their area under curve (AUC) were used to assess the predictive power. Subgroup analyses were conducted in patients with AFL and patients with ALC, and MDF and MELD were used for prognosis evaluation. ML models and nomogram were performed using EmpowerStats and R software, and other analyses were carried out in STATA 15.0 software (Stata Corp, College Station, TX, USA). *P* < 0.05 was considered statistically significant.

**References**

1. Zhang Y, Guo T, Yang F, Mao Y, Li L, Liu C, Sun Q, et al. Single-nucleotide rs738409 polymorphisms in the PNPLA3 gene are strongly associated with alcoholic liver disease in Han Chinese males. Hepatol Int 2018;12:429-437.

2. Chang D, Truong E, Mena EA, Pacheco F, Wong M, Guindi M, Todo TT, et al. Machine learning models are superior to noninvasive tests in identifying clinically significant stages of NAFLD and NAFLD-related cirrhosis. Hepatology 2023;77:546-557.

3. Niu L, Thiele M, Geyer PE, Rasmussen DN, Webel HE, Santos A, Gupta R, et al. Noninvasive proteomic biomarkers for alcohol-related liver disease. Nat Med 2022;28:1277-1287.

4. Liu SY, Tsai IT, Hsu YC. Alcohol-Related Liver Disease: Basic Mechanisms and Clinical Perspectives. Int J Mol Sci 2021;22.

5. Wang H, Mehal W, Nagy LE, Rotman Y. Immunological mechanisms and therapeutic targets of fatty liver diseases. Cell Mol Immunol 2021;18:73-91.

6. Ren R, He Y, Ding D, Cui A, Bao H, Ma J, Hou X, et al. Aging exaggerates acute-on-chronic alcohol-induced liver injury in mice and humans by inhibiting neutrophilic sirtuin 1-C/EBPα-miRNA-223 axis. Hepatology 2022;75:646-660.

7. Chauhan A, Adams DH, Watson SP, Lalor PF. Platelets: No longer bystanders in liver disease. Hepatology 2016;64:1774-1784.

Supplementary **Table 1. Characteristics of patients with ALD and healthy controls**

|  | HC (n=234) | ALD (n=207) | | | AFL vs. HC | | ALC vs. HC | | ALD vs. HC | |
| --- | --- | --- | --- | --- | --- | --- | --- | --- | --- | --- |
|  |  | AFL (n=99) | ALC (n=108) | Total (n=207) | χ^2^/t/Z | *P* | χ^2^/t/Z | *P* | χ^2^/t/Z | *P* |
| Gender (male, %) | 194 (82.9) | 96 (96.9) | 105 (97.2) | 201 (97.1) | 12.236^*^ | 0.001 | 13.778^*^ | <0.001 | 23.690^*^ | <0.001 |
| Age (years) | 52.1±12.9 | 50.8±10.5 | 58.8±10.3 | 55.0±11.1 | 0.864^#^ | 0.348 | -5.167^#^ | <0.001 | -2.535^#^ | 0.012 |
| Basophils (x10^9/L) | 0.03 (0.02, 0.05) | 0.02 (0.01, 0.04) | 0.01 (0.01, 0.02) | 0.01 (0.01, 0.03) | -5.418 | <0.001 | -9.551 | <0.001 | -9.326 | <0.001 |
| Eosinophils (x10^9/L) | 0.12 (0.07, 0.20) | 0.09 (0.05, 0.15) | 0.06 (0.03, 0.10) | 0.07 (0.03, 0.12) | -2.731 | 0.006 | -7.257 | <0.001 | -6.260 | <0.001 |
| MCV (fl) | 44.4±3.7 | 29.7±7.9 | 27.4±7.7 | 28.5±7.9 | 17.678^#^ | <0.001 | 21.707^#^ | <0.001 | 26.522^#^ | <0.001 |
| Lymphocyte(x10^9/L) | 2.0 (1.5, 2.4) | 0.9 (0.5, 1.7) | 0.7 (0.5, 1.1) | 0.8 (0.5, 1.3) | -8.948 | <0.001 | -11.953 | <0.001 | -12.985 | <0.001 |
| Monocyte (x10^9/L) | 0.4 (0.3, 0.5) | 0.3 (0.2, 0.5) | 0.3 (0.2, 0.5) | 0.3 (0.2, 0.5) | -2.959 | 0.003 | -3.285 | 0.001 | -3.869 | <0.001 |
| Neutrophil (x10^9/L) | 3.7 (2.9, 4.5) | 1.9 (1.1, 2.7) | 2.3 (1.5, 4.0) | 2.1 (1.4, 3.2) | -10.338 | <0.001 | -6.742 | <0.001 | -10.505 | <0.001 |
| Platelet (x10^9/L) | 240 (196, 281) | 81(48, 152) | 67 (42, 103) | 71 (44, 119) | -11.769 | <0.001 | -13.405 | <0.001 | -15.614 | <0.001 |
| RBC (x10^12/L) | 5.0±0.5 | 3.5±0.9 | 3.1±0.8 | 3.3±0.9 | 15.626^#^ | <0.001 | 22.815^#^ | <0.001 | 24.992^#^ | <0.001 |
| WBC (x10^9/L) | 6.5±1.8 | 3.8±1.9 | 4.4±2.7 | 4.1±2.4 | 12.128^#^ | <0.001 | 7.221^#^ | <0.001 | 11.573^#^ | <0.001 |
| ALT (u/L) | 22 (17, 30) | 27 (19, 41) | 27 (19, 83) | 27 (19, 44) | -3.037 | 0.002 | -2.980 | 0.003 | -3.722 | <0.001 |
| AST (u/L) | 21 (19, 25) | 34 (28, 48) | 42 (29, 83) | 36 (29, 60) | -9.976 | <0.001 | -10.856 | <0.001 | -12.899 | <0.001 |
| AST/ALT | 1.0 (0.7, 1.2) | 1.2 (0.9, 1.6) | 1.6 (1.3, 2.1) | 1.4 (1.1, 1.9) | -5.739 | <0.001 | -10.295 | <0.001 | -10.000 | <0.001 |
| ALP (u/L) | 70.8±19.2 | 122.9±61.9 | 135.2±71.8 | 129.3±67.3 | -8.038^#^ | <0.001 | -9.021^#^ | <0.001 | -11.651^#^ | <0.001 |
| γ-GT (u/L) | 26 (17, 45) | 63 (37, 129) | 79 (40, 181) | 73 (39, 148) | -8.142 | <0.001 | -8.031 | <0.001 | -9.063 | <0.001 |
| PMR | 628 (502, 797) | 292 (179, 378) | 230 (132, 331) | 249 (162, 359) | -11.660 | <0.001 | -12.840 | <0.001 | -15.180 | <0.001 |
| MLR | 0.2 (0.1, 0.3) | 0.3 (0.2, 0.4) | 0.4 (0.3, 0.5) | 0.3 (0.2, 0.5) | -9.238 | <0.001 | -11.077 | <0.001 | -12.602 | <0.001 |
| MNR | 0.1 (0.09, 0.12) | 0.2 (0.1, 0.2) | 0.1 (0.1, 0.2) | 0.14 (0.10, 0.19) | -8.371 | <0.001 | -4.788 | <0.001 | -8.078 | <0.001 |
| PLR | 124 (97, 152) | 83 (62, 133) | 94 (55, 135) | 88 (62, 133) | -5.129 | <0.001 | -5.123 | <0.001 | -6.343 | <0.001 |
| NLR | 1.9 (1.5, 2.5) | 1.9 (1.3, 3.1) | 3 (2, 5) | 2.4 (1.6, 4.1) | -0.440 | 0.660 | -7.384 | <0.001 | -4.963 | <0.001 |
| PNR | 65 (51, 81) | 46 (26, 66) | 28 (18, 42) | 36 (21, 52) | -5.795 | <0.001 | -11.519 | <0.001 | -10.813 | <0.001 |

*, χ^2^ value; #, t vaule. ALD, alcoholic liver disease; ALC, alcoholic liver cirrhosis; AFL, alcoholic fatty liver; ALT, alanine transaminase; AST, aspartate transaminase; ALP, alkaline phosphatase; HC, Healthy controls; MCV, mean corpuscular volume; MLR, monocyte/lymphocyte ratio; MNR, monocyte/neutrophil ratio; NLR, neutrophil/lymphocyte ratio; PLR, platelet/lymphocyte ratio; PNR, platelet/neutrophil ratio; RBC, red blood cell; WBC, white blood cell; γ-GT, gamma-glutamyl transferase.

Supplementary **Table 2. Evaluation of machine learning models**

|  | RMSE | | MSE | |
| --- | --- | --- | --- | --- |
|  | Train | Test | Train | Test |
| **ALD onset** | |  |  |  |
| **ALD vs. HC** |  |  |  |  |
| MLP | 0.1048 | 0.0954 | 0.0110 | 0.0091 |
| RF | 0.1785 | 0.1194 | 0.0319 | 0.0143 |
| GBM | 0.0251 | 0.0962 | 0.0006 | 0.0092 |
| GLM | 0.1320 | 0.0981 | 0.0174 | 0.0096 |
| **ALD progression** |  |  |  |  |
| **AFL vs. HC** |  |  |  |  |
| MLP | 0.0631 | <0.0001 | 0.0040 | <0.0001 |
| RF | 0.1445 | 0.0618 | 0.0209 | 0.0038 |
| GBM | 0.0049 | 0.0006 | <0.0001 | <0.0001 |
| GLM | 0.1121 | 0.0323 | 0.0126 | 0.0010 |
| **ALC vs. HC** |  |  |  |  |
| MLP | 0.1349 | 0.1055 | 0.0182 | 0.0111 |
| RF | 0.1971 | 0.1522 | 0.0388 | 0.0232 |
| GBM | 0.0499 | 0.1131 | 0.0025 | 0.0128 |
| GLM | 0.1397 | 0.1261 | 0.0195 | 0.0159 |
| **ALC vs. AFL** |  |  |  |  |
| MLP | 0.5280 | 0.4945 | 0.2788 | 0.2445 |
| RF | 0.4490 | 0.4595 | 0.2016 | 0.2112 |
| GBM | 0.3608 | 0.4457 | 0.1302 | 0.1986 |
| GLM | 0.4151 | 0.4675 | 0.1723 | 0.2185 |
| **ALD prognosis** | |  |  |  |
| **MDF (<32.2 vs. ≥32.2)** | | | |  |
| MLP | 0.3006 | 0.4510 | 0.0904 | 0.2034 |
| RF | 0.5192 | 0.4036 | 0.2695 | 0.1629 |
| GBM | 0.4279 | 0.4223 | 0.1831 | 0.1784 |
| GLM | 0.4471 | 0.4390 | 0.1999 | 0.1927 |
| **MELD (<20 vs. ≥20)** | | | |  |
| MLP | 0.2524 | 0.3432 | 0.0637 | 0.1178 |
| RF | 0.3172 | 0.3352 | 0.1006 | 0.1124 |
| GBM | 0.1710 | 0.3464 | 0.0293 | 0.1200 |
| GLM | 0.2604 | 0.3499 | 0.0678 | 0.1224 |

ALD, alcoholic liver disease; ALC, alcoholic liver cirrhosis; AFL, alcoholic fatty liver; MLP, multilayer perceptron; GBM, gradient boosting machine; GLM, generalized linear model; HC, Healthy control; MSE, mean square error; RMSE, root mean square error; RF, random forest.


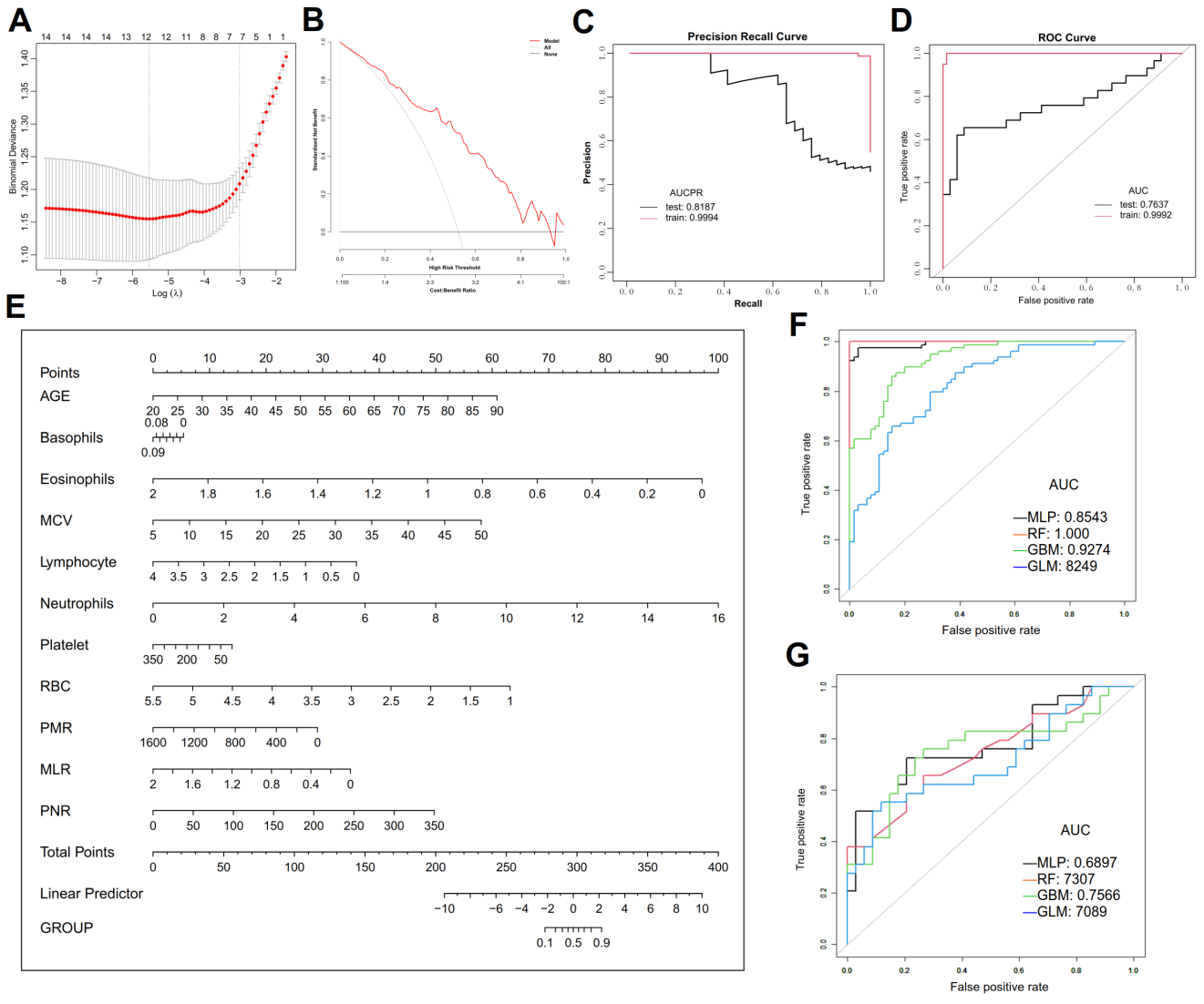


**SFigure 1. LASSO regression, nomogram, and ML performance in the estimation of AFL and ALC.**

**(A)** LASSO regression profiles of 11 parameters. **(B)** DCA curve for assessing the predictive ability and accuracy of the nomogram. **(C)** AUCPR of the best ML model. **(D)** AUCs of the best ML model. **(E)** Nomogram for predicting probability. **(F)** AUCs of the four ML model in the training set. **(G)** AUCs of the four ML model in the testing set.
